# Role of artificial intelligence in staging and assessing of treatment response in MASH patients

**DOI:** 10.3389/fmed.2024.1480866

**Published:** 2024-10-21

**Authors:** Reha Akpinar, Davide Panzeri, Camilla De Carlo, Vincenzo Belsito, Barbara Durante, Giuseppe Chirico, Rosa Lombardi, Anna Ludovica Fracanzani, Marco Maggioni, Ivan Arcari, Massimo Roncalli, Luigi M. Terracciano, Donato Inverso, Alessio Aghemo, Nicola Pugliese, Laura Sironi, Luca Di Tommaso

**Affiliations:** ^1^Department of Pathology, IRCCS Humanitas Research Hospital, Milan, Italy; ^2^Department of Biomedical Sciences, Humanitas University, Milan, Italy; ^3^Department of Physics, Università di Milano-Bicocca, Milan, Italy; ^4^SC Medicina Indirizzo Metabolico, Fondazione IRCCS Ca' Granda Ospedale Maggiore Policlinico, Milan, Italy; ^5^Department of Pathophysiology and Transplantation, University of Milan, Milan, Italy; ^6^Division of Pathology, Foundation IRCCS Ca’ Granda Ospedale Maggiore Policlinico, Milan, Italy; ^7^Division of Internal Medicine and Hepatology, Department of Gastroenterology, IRCCS Humanitas Research Hospital, Rozzano, Italy; ^8^Division of Immunology, Transplantation and Infectious Diseases IRCCS San Raffaele Scientific Institute, Vita-Salute San Raffaele University, Milan, Italy

**Keywords:** liver, MASH, fibrosis, treatment, artificial intelligence

## Abstract

**Background and Aims:**

The risk of disease progression in MASH increases proportionally to the pathological stage of fibrosis. This latter is evaluated through a semi-quantitative process, which has limited sensitivity in reflecting changes in disease or response to treatment. This study aims to test the clinical impact of Artificial Intelligence (AI) in characterizing liver fibrosis in MASH patients.

**Methods:**

The study included 60 patients with clinical pathological diagnosis of MASH. Among these, 17 received a medical treatment and underwent a post-treatment biopsy. For each biopsy (n = 77) a Sirius Red digital slide (SR-WSI) was obtained. AI extracts >30 features from SR-WSI, including estimated collagen area (ECA) and entropy of collagen (EnC).

**Results:**

AI highlighted that different histopathological stages are associated with progressive and significant increase of ECA (F2: 2.6% ± 0.4; F3: 5.7% ± 0.4; F4: 10.9% ± 0.8; p: 0.0001) and EnC (F2: 0.96 ± 0.05; F3: 1.24 ± 0.06; F4: 1.80 ± 0.11, p: 0.0001); disclosed the heterogeneity of fibrosis among pathological homogenous cases; revealed post treatment fibrosis modification in 76% of the cases (*vs* 56% detected by histopathology).

**Conclusion:**

AI characterizes the fibrosis process by its true, continuous, and non-categorical nature, thus allowing for better identification of the response to anti-MASH treatment.

## 1 Introduction

Metabolic Dysfunction-Associated Steatotic Liver Disease (MASLD) is the most common cause of chronic liver disease worldwide, with a global prevalence of 25% in the adult population, 60% in patients with type 2 diabetes and 80% in obese individuals (1, 2). Only a small proportion of MASLD cases progress to cirrhosis and severe complications, but biomarkers predicting patients at risk are still lacking. Conversely, in patients where steatosis is complicated by inflammation and cellular damage (i.e., MASH - Metabolic Dysfunction-Associate Steatohepatitis), the possibility of severe liver diseases increases proportionally with the amount of fibrosis, as estimated by the pathological stage (3, 4). In keeping with this, the histopathological reduction of fibrosis is one of the main endpoints for clinical trials on MASH patients (5–7). However, the histopathological assessment of fibrosis stage in chronic liver disease, including MASH (8, 9), is a descriptive and semi-quantitative process with moderate reproducibility even among expert liver pathologists (10, 11). Moreover, the limited categories used for pathological staging scarcely illustrate a disease driven by a continuum of injury, resulting in limited sensitivity to reflect changes in disease severity over time (11).

Approaches based on whole slide digital images (WSI) and Artificial Intelligence (AI) have been applied successfully to the evaluation of morphological features of MASH, including fibrosis (12–18). Notably, two methods have shown clinically meaningful results. Collagen Proportionate Area (CPA), a quantitative assessment of collagen area expressed as the ratio of collagen-stained pixels over full-biopsy-pixels, demonstrated its superiority over semi-quantitative staging in predicting clinical decompensation (19, 20). Second Harmonic Generation (SHG) analysis, which focuses on the possibility to visualize collagen allowing its quantification on a continuous scale and evaluation of spatial relationship with the surrounding cells (14, 21), demonstrated its superiority over pathological staging in detecting treatment-associated reduction of fibrosis (15). These new methods, allowing to characterize minimal difference in the quantitative features of the fibrous tissue, pave the way to precision medicine also in hepatology (11).

The aim of this study is to test the clinical impact of an AI-based approach in characterizing liver fibrosis features in a series of MASH patients.

## 2 Materials and methods

### 2.1 Study cohort

We searched the files of the Departments of Hepatology and Pathology of IRCCS, Humanitas Clinical Institute (Rozzano, Italy), for all cases with a matched clinical and pathological diagnosis of MASH. Specifically, we selected only cases fulfilling the following criteria: (1) clinical diagnosis of MASLD or MetALD (MASLD predominant), (2) histopatological diagnosis of steato-hepatitis (8, 9); (3) biopsy tissue of >15 mm length, showing >10 portal spaces.

### 2.2 Adjudication of ground truth

For each biopsy, we obtained a recut and stained all of them in a single batch for Sirius Red (SR). All the SR slides were then digitized using Ultra Fast Scanner (Philips, Netherlands). Imaging was performed with an Olympus 40x air objective (NA = 0.75, Plan Apo) with a pixel-size of 0.25 𝜇m.

SR digital slides (SR-WSI) were then evaluated using the Philips viewer independently by four pathologists (two senior, expert in liver pathology; two young, without specific experience in liver pathology). Each of them evaluated the pathological stage according to the CNR NASH semi-quantitative system (8, 9). Discordant diagnoses were reviewed in a consensus session.

Inter-observer variability was evaluated using Fleiss’s kappa value, *κ* (22, 23). Kappa values indicate slight agreement when the value ranges between 0.01 and 0.20, fair agreement (0.21–0.40), moderate agreement (0.41–0.60), substantial agreement (0.61–0.80), and almost perfect agreement when the values are >0.81. This process allowed to quantify inter-observer concordance and to establish a conclusive microscopic-stage for each case (ground truth).

### 2.3 AI-based analysis

Once labeled with a conclusive histopathological stage, each SR-WSI underwent an AI-based analysis to carefully measure several fibrosis features (see Supplementary material for details). Dependent t-test (called the paired-samples t-test) was then used in order to assess differences for each fibrosis feature. Statistical significance has been calculated for all paired categories and considered significant when *p*-value less than 0.05. This approach allowed to explore differences existing in the fibrosis features in cases labeled with the same histopathological stage.

### 2.4 Assessment of treatment effect: histopathology versus AI

We then focused our attention on a subset of cases from the study series, specifically those who received a liver biopsy before and after medical therapy for MASH within a clinical trial. In particular, we compared the impact of AI versus histopathology in assessing changes in fibrosis, specifically the increase or reduction of fibrosis, to evaluate the treatment effect in paired biopsy.

### 2.5 Validation

To validate the clinical findings emerging from the study cohort, particularly the impact of AI in assessing an increase or reduction of fibrosis, we collected an adequate external series. We searched the files of the Departments of Hepatology and Pathology at IRCCS, Ca′ Granda, Ospedale Maggiore Policlinico (Milano, Italy) for cases fulfilling the following criteria: (1) clinical diagnosis MASLD or metALD (MASLD predominant); (2) histopatological diagnosis of steatohepatitis (8, 9); (3) biopsy tissue of >15 mm length, showing >10 portal spaces; (4) liver biopsy obtained before and after medical treatment within a clinical trial for MASH. Once the cases were selected and recuts obtained, all samples were stained in a single batch for Sirius red (SR) at the original Institution. All the SR slides were then digitized using the same scanner of the study cohort. Once generated, SR-WSIs were evaluated by the same AI solution used in the study cohort. In the validation cohort, the original pathological stage, based on CNR NASH semi-quantitative system, evaluated by an expert liver pathologist, was considered as truth.

### 2.6 Assessment of clinical outcomes

We assessed clinical outcomes in terms of weight loss. A clinical outcome was defined as a weight reduction of more than 10% between the baseline biopsy and the follow-up biopsy, based on its known association with improvements in liver histology in terms of liver fibrosis in MASH patients (24). This criterion was applied to both the study and validation cohorts. Written informed consent was obtained from the individuals for the publication of any potentially identifiable images or data included in this article.

## 3 Results

**Figure 1:**
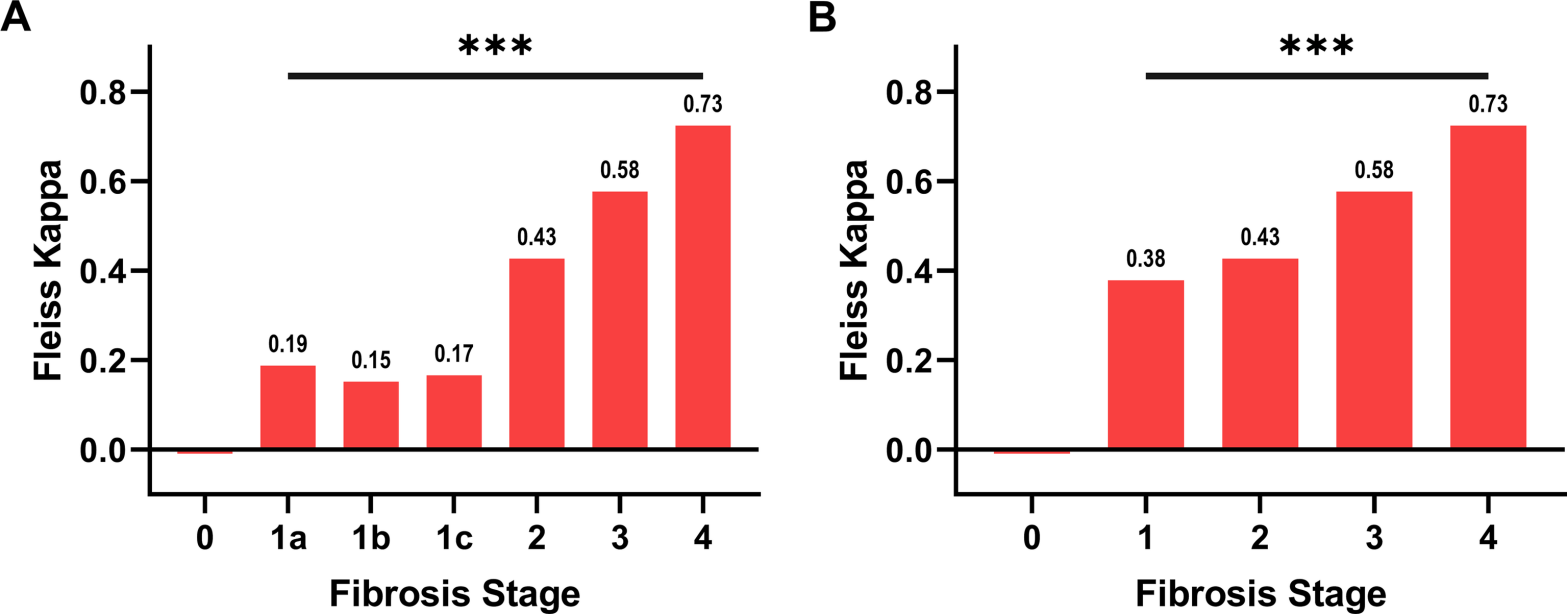
Agreement between pathologists in the evaluation of NASH stage. In panel (A), the subgroups of F1, namely F1a, F1b, F1c, have been considered separately, while in panel (B) they have been grouped. The overall Fleiss’s Kappa agreement is 0.44 for panel A and 0.55 for panel B. The agreement for each category is reported over the column. The hypothesis that the agreement is caused by random chance can be rejected (*** *p* ≤ 0.001).

The study cohort consisted of 52 patients (Table 1). Among them, 9 underwent a post-treatment biopsy. Therefore, the total number of cases was 61. The results of the pathologists’ agreement in staging these cases are shown in Figure 1. The agreement was fair for F1a, F1b and F1c (respectively *κ*: 0.19, 0.15 and 0.17), moderate for F2 (κ: 0.43) and F3 (κ: 0.58) and substantial for F4 (κ: 0.73). The overall agreement was only moderate (κ: 0.44 Figure 1A, rising to κ: 0.55 when grouping all F1 classes into a single category, Figure 1B). After the adjudication, the series was represented by 9 F1 (a,b,c: 4,1,4), 19 F2, 28 F3 and 5 F4. These diagnoses were then considered as the ground truth.

**Table 1:** Demographic and clinical features of the study cohort (*n* = 52).

	All patients*
	(*n* = 52)
Age, mean (std)	56.6 (10.4)
Male sex, *n* (%)	33 (63.4)
Ethnicity, *n* (%)	
Caucasian	51 (98%)
Hispanic	1 (2%)
Body mass index (kg/m^2^), mean(std)	29.2 (4.7)
Overweight, *n* (%)	21 (40%)
Obesity, *n* (%)	20 (38.4%)
Hypertension, *n* (%)	22 (42.3)
DM, *n* (%)	9 (17.3)
Dyslipidemia, *n* (%)	22 (42.3)
MASLD, *n* (%)	49 (94.2%)
MetALD, *n* (%)	3 (5.8%)
MASLD predominant, *n* (%)	3 (5.8%)
Liver-related events, *n* (%)	0
Extrahepatic events, *n* (%)	0

std, standard deviation; DM, diabetes mellitus; MASLD, Metabolic Dysfunction Associated Steatotic Liver Disease; MetALD, Metabolic alcohol-related fatty liver disease.

### 3.1 Homogeneous histopathological stages are finely dissected by AI-based analysis

**Figure 2:**
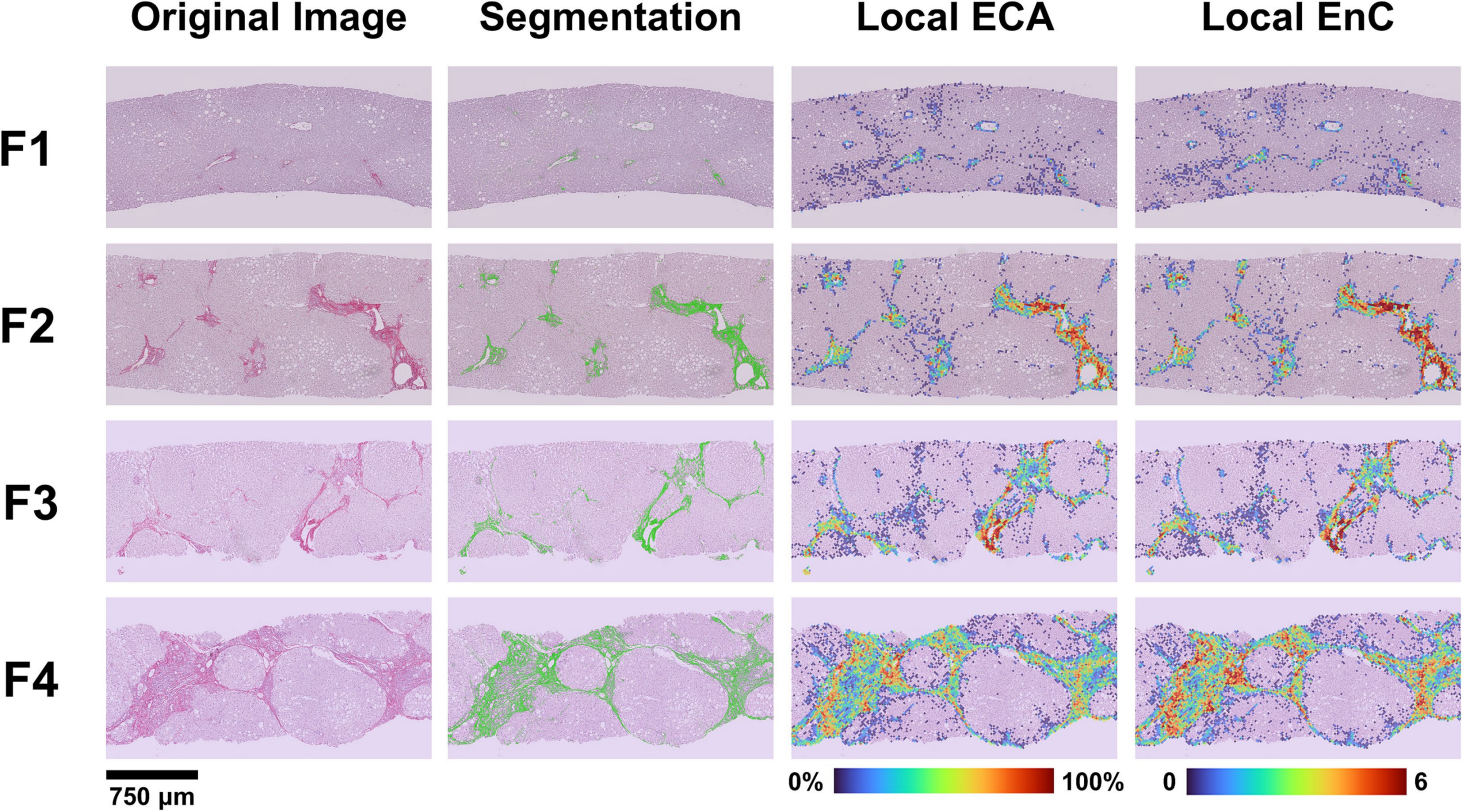
Correlation between histopathological NASH stage and features of fibrosis generated by AI. The figure illustrates the process of transition from the original image (SR-WSI) to AI-features and the comparison of the histopathological NASH stage (F1 to F4) to a heatmap generated by the AI for ECA and EnC. After preprocessing (see Supplementary Figure S1), the original SR-WSI is analyzed to segment collagen. The quantification process involves the extraction of both intensity and textural features at pixel level and within Regions of Interest (ROIs). Estimated Collagen Area (ECA) is computed as a fraction of collagen pixels (Sirius Red positive) over the total number of pixels representing the tissue section. Entropy of Collagen (EnC) is a textural parameter that encodes for the randomness of SR optical density values with respect to its neighborhood in terms of intensity distribution. Low entropy values correspond to a uniform and homogeneous image.

The AI-based analysis finely dissected the histopathological category of “fibrosis” into several parameters (n = 33, see Supplementary Table S1 for a detailed list). Significant differences along the spectrum F1-F4 were observed for 14 parameters (see Supplementary Table S2 for details). Among these latter, we focused our interest on Estimated Collagen Area (ECA) and Entropy of Collagen (EnC), since both have an easy reference to a morphological counterpart. Figure 2 illustrates and explains the transition from histopathological stage to ECA and EnC.

#### 3.1.1 Estimated collagen area compared to microscopic stages

**Figure 3:**
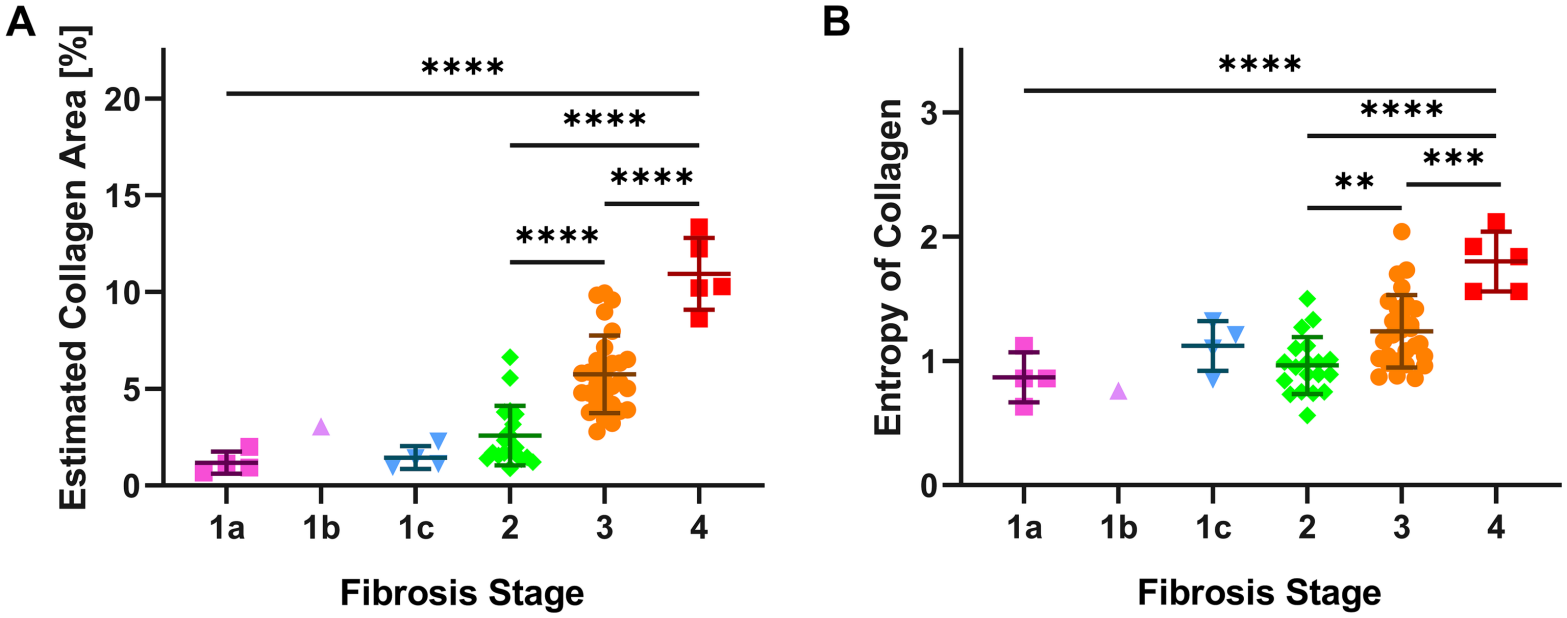
AI features (ECA and EnC) of single cases according to histopathological NASH stage. The figure shows for each biopsy, grouped according to the histopathological stage, the results of AI evaluation. Panel (A) illustrates the Estimated Collagen Area (ECA), panel (B) describes the evaluation of Entropy of Collagen (EnC). Significant differences between subgroups are assessed by one-way ANOVA corrected by a Tukey *post hoc* test. (** *p* ≤ 0.01; *** p ≤ 0.001; **** *p* ≤ 0.0001). Error bars represent the standard error of mean.

**Figure 4:**
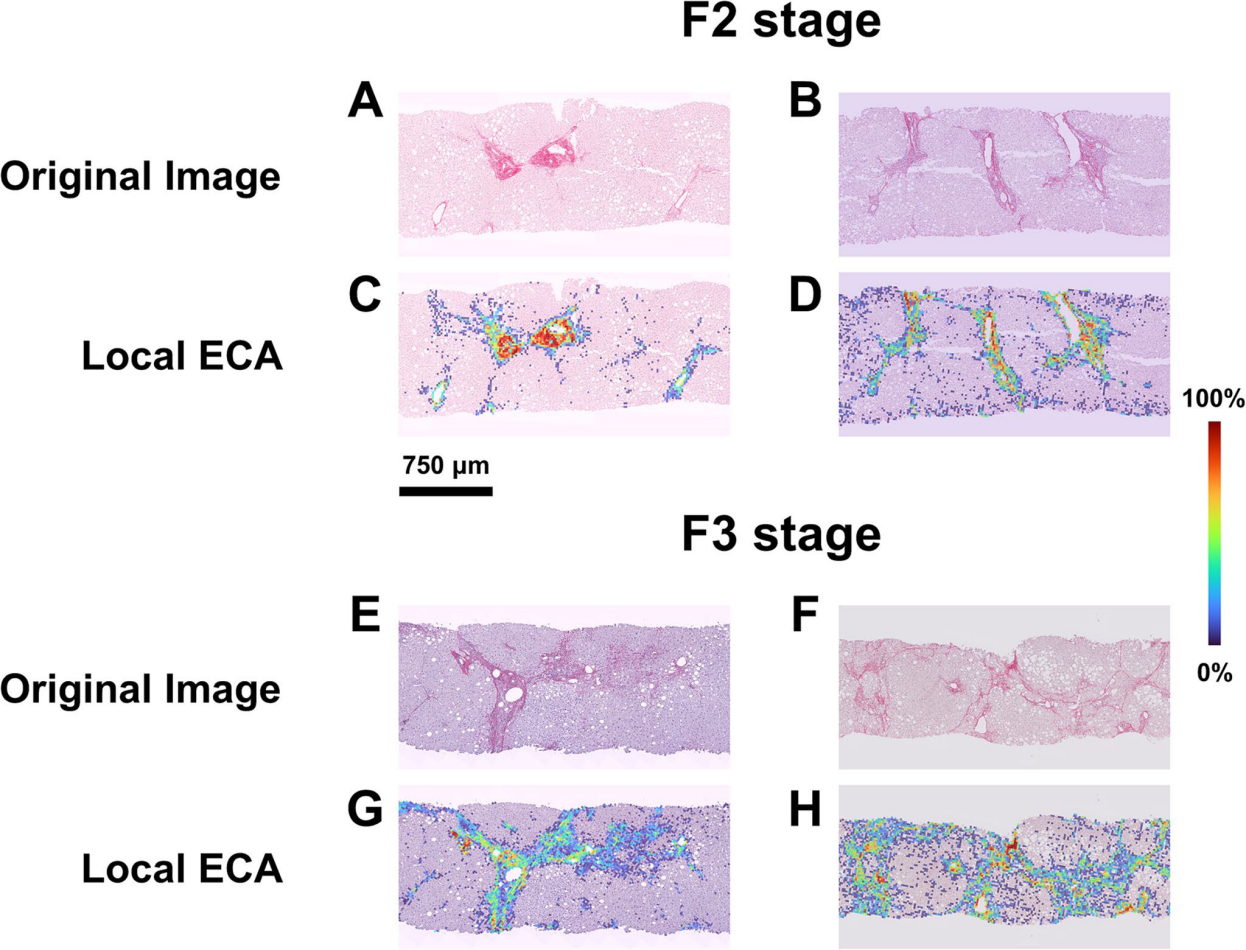
ECA heterogeneity in cases with homogenous histopathological NASH stage. (A,B) SR-WSI of two cases labeled as F2 at histopathological level by all pathologists (perfect agreement). (C,D) Overlay between the original WSI and the Estimated Collagen Area (ECA) heatmap for each case. AI shows that in the first case (heatmap C) the percentage of tissue area covered by collagen (ECA) is 1.67% while in the second case (heatmap D) this percentage is to 3.85%. (E,F) SR-WSI of two cases labeled as F3 at histopathological level, after the adjudication process. (G,H) AI computes, that the area covered by collagen is lower in the first case (heatmap G, ECA = 3.83%) as compared to the second (heatmap H, ECA = 9.95%).

The mean values of ECA were progressively higher and significantly different in F2, F3 and F4 categories (Figure 3A; Supplementary Table S3). Interestingly, ECA of individual cases within the same histopathological stage showed considerable heterogeneous values; some values overlapped with those of cases in adjacent stages. This heterogeneity characterized in particular F3 cases, where ECA ranged between 2.8 and 10% (mean 5.7%, standard deviation ±0.4%, IQR 2.3). The heterogeneity of ECA in two exemplificative cases is shown in Figure 4.

#### 3.1.2 Entropy of collagen compared to microscopic stages

EnC also showed a statistically significant increase from F2 to F4 (Figure 3B; Supplementary Table S4), with considerable heterogeneity of values within homogenous pathological categories. In particular, F3 cases exhibited EnC levels ranging between 0.86 and 2.04 (mean: 1.24; standard deviation: ±0.06; IQR: 0.4), while F4 cases ranged between 1.56 and 2.12 (mean: 1.8; standard deviation ±0.1; IQR: 0.5). Supplementary Figure S2 illustrates the heterogeneity of EnC in two exemplificative cases.

### 3.2 AI-based analysis highlights treatment efficacy

In the study cohort, 9 patients underwent a liver biopsy before and after medical treatment for MASH (see Supplementary Table S5a for clinical data). We compared the ability of histopathology (stage) and AI (ECA and EnC) to recognize changes due to therapy. The results are shown in Table 2. Briefly, 56% of cases showed a stage change according to pathologist-based evaluation, compared to 100% depicted by AI. According to AI analysis, all post treatment biopsies differed from their paired pretreatment counterparts in terms of ECA and EnC. Among cases without changes at the microscopic level, 75% were characterized by a homogenous decrease of ECA and EnC, while the remaining 25% had homogeneous increase in both AI values. Among cases that showed a stage change at the microscopic level, 80% had congruous modifications in both ECA and EnC; in the remaining 20%, ECA and EnC were congruous but opposite to the histopathological finding. At clinical level two patients (n° 2 and 6) achieved a weight loss greater than 10% between the baseline and post-treatment biopsy; in both cases, AI demonstrated a congruous reduction in ECA and EnC while histopathology recognize a change of stage (reduction) only in one case.

**Table 2:** Histopathological stage, ECA and EnC in pre- and post- treatment biopsy (study cohort).

Case	Histopathological stage	ECA (%)	EnC
1 pre	4	8.6%	1.56
1 post	3	6.3%	1.38
2 pre	3	5.8%	0.87
2 post	2	3.9%	0.84
3 pre	3	6.5%	1.16
3 post	2	6.6%	1.33
4 pre	3	9.6%	1.48
4 post	3	8.0%	1.42
5 pre	1a	2.0%	0.86
5 post	2	2.7%	0.95
6 pre	3	9.8%	2.04
6 post	3	4.8%	1.42
7 pre	3	5.8%	1.08
7 post	3	5.9%	1.29
8 pre	3	3.2%	1.25
8 post	4	13.4%	1.92
9 pre	3	5.2%	1.04
9 post	3	5.0%	0.98

In the validation cohort, there were 8 patients who received liver biopsy before and after medical treatment for MASH (see Supplementary Table S5b for clinical data). Changes in pathological stage, ECA and EnC observed in these patients are reported in Table 3. Similar to the study cohort, histopathological evaluation disclosed stage change after treatment in 50% of cases as compared to 100% detected by AI. Among cases without changes at microscopic level, 50% were characterized by a clear and homogenous decrease of both ECA and EnC. Among cases with a proven change of stage at histopathology, 50% had congruous modifications of both ECA and EnC. In the validation cohort, no patient achieved the >10% weight loss threshold.

**Table 3:** Histopathological stage, ECA and EnC in pre- and post- treatment biopsy (validation cohort).

Case	Histopathological Stage	ECA (%)	EnC
1 Pre	2	3.48%	0.99
1 Post	3	5.06%	0.74
2 Pre	3	5.75%	1.94
2 Post	3	5.00%	1.69
3 Pre	3	5.18%	1.66
3 Post	2	5.09%	1.46
4 Pre	1a	1.33%	0.67
4 Post	2	3.34%	0.60
5 Pre	3	6.17%	0.69
5 Post	3	2.04%	1.21
6 Pre	3	6.10%	1.86
6 Post	3	1.55%	0.90
7 Pre	3	3.86%	1.48
7 Post	3	4.13%	0.75
8 Pre	4	9.03%	1.64
8 Post	2	1.87%	0.74

**Figure 5:**
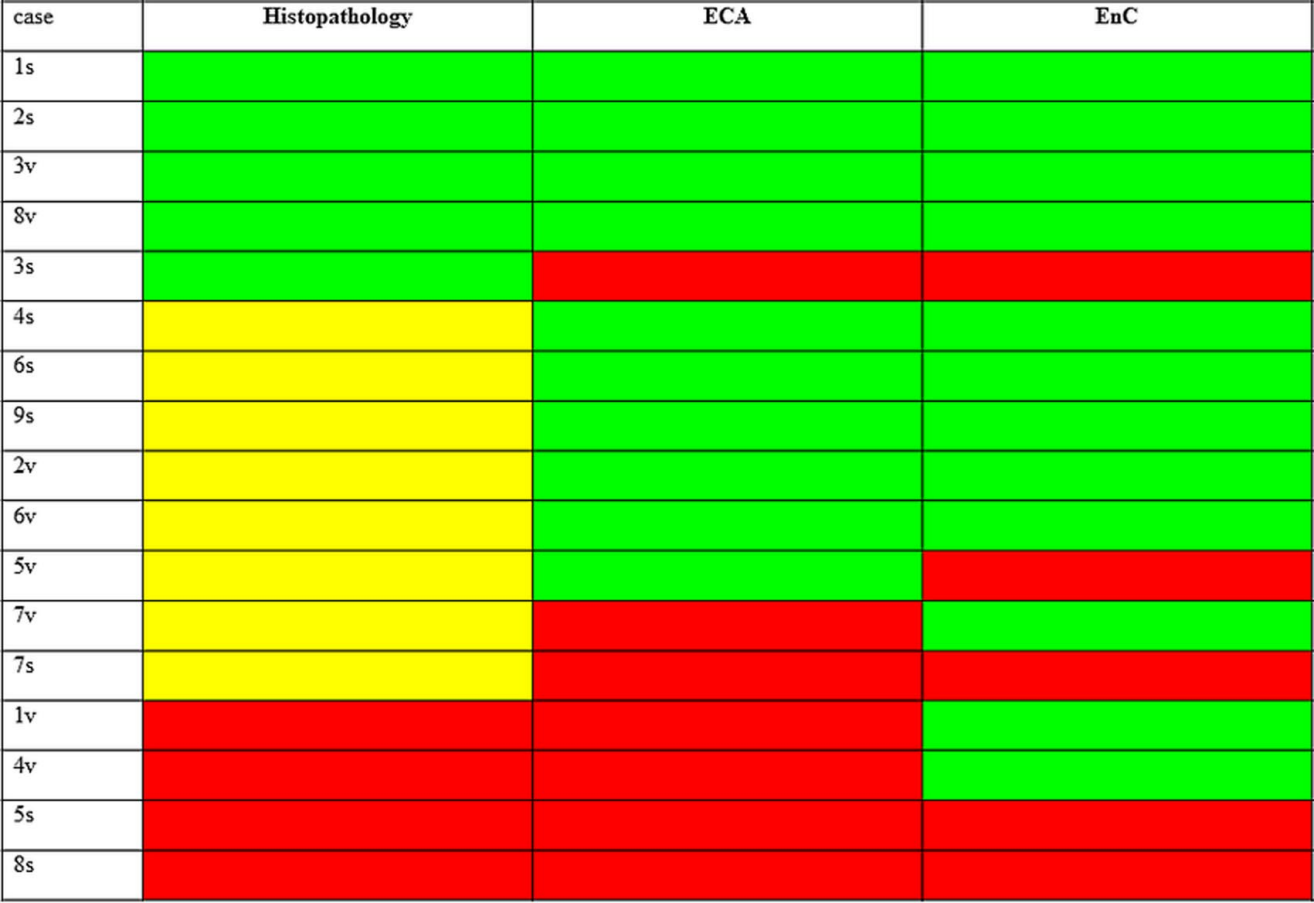
Impact of AI, as compared to histopathology, in the assessment of treatment efficacy taking as reference the post treatment biopsy. The figure compares the results of histopathology and AI in assessing the response to treatment for MASH. Results shown refers to post treatment biopsy (PTB). Histopathology: green = reduction of stage in PTB (responder at histopathology); yellow = no change of stage in PTB (not conclusive at histology); red = increase of stage in PTB (not responder at histopathology). AI (ECA and EnC): green = reduction of value in PTB; red = increase of value in PTB; cases with congrous decrease of ECA and EnC are responders, with congrous decrease not responders, and discordant case as not conclusive, according to AI analysis.

**Figure 6:**
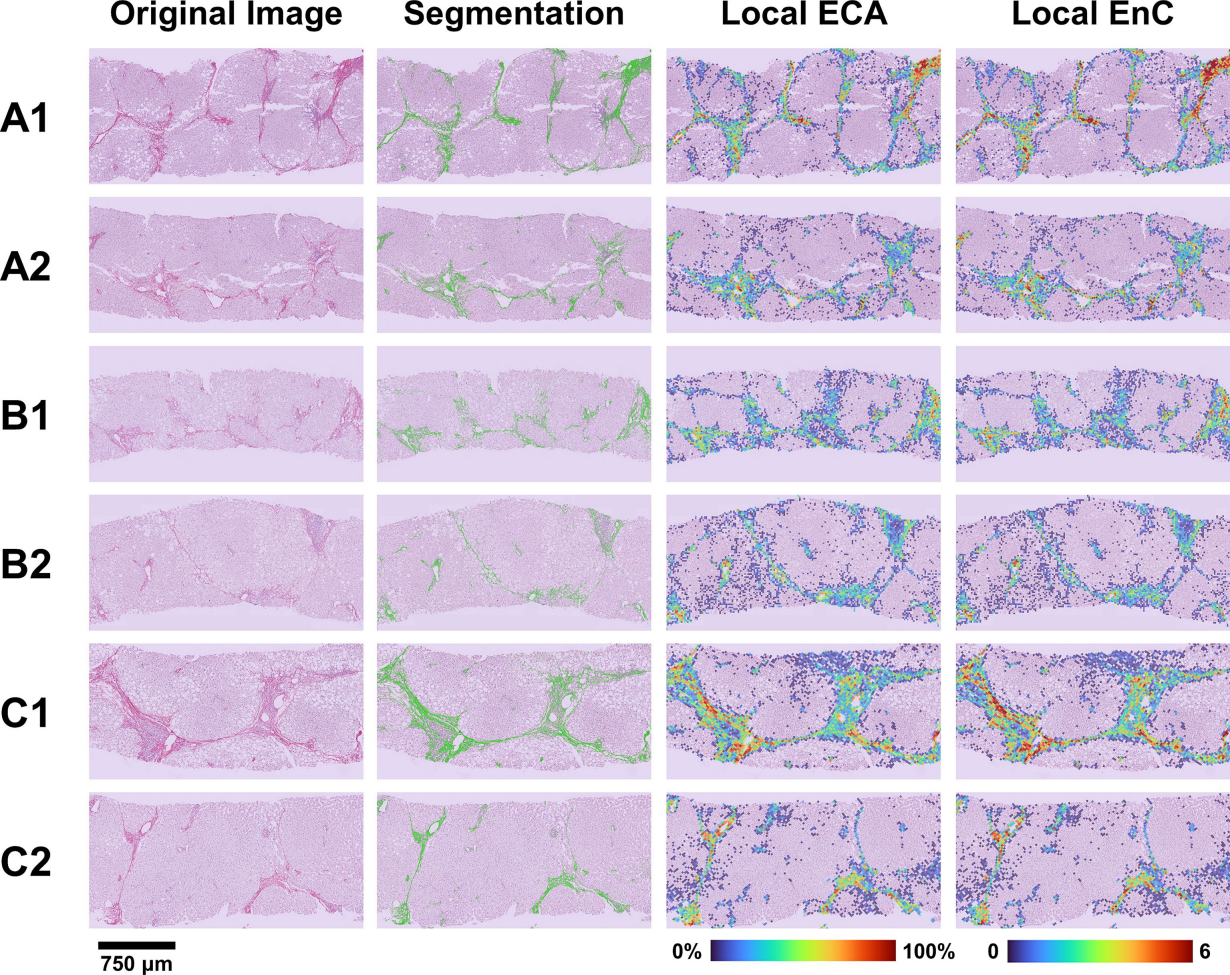
Evaluation of treatment efficacy on fibrosis modification. The figure illustrates the microscopic (SR-WSI, left) and AI (segmentation, ECA and EnC, right) features in pre- and post- treatment biopsy in three patients (A, B, C). In patient A (case 6, Table 2), the pre treatment biopsy (A1) was diagnosed as stage F3, AI reveled a ECA of 9.8% and EnC of 2.04; post treatment biopsy (A2) was still diagnosed as F3 at histopathological level; however AI revealed that EC decreased to 4.8% and EnC to 1.42. In patient B (case 4, Table 2), the pre treatment biopsy (B1) was diagnosed as F3, with ECA of 9.6% and EnC of 1.48; after treatment (B2) the histopathological stage did not change (F3), but AI disclosed a reduction for both ECA (8.0%) and EnC (1.42). In Patient C (case 1, Table 2), the histopathological evaluation disclosed a stage reduction from F4 seen in pre-treatment (C1) to F3 seen in post treatment biopsy (C2); AI features were consistent with this reduction (ECA from 8.6 to 6.3%; EnC from 1.56 to 1.38).

The heatmap shown in Figure 5 offers an overview of the impact of AI, compared to histopathology, in the assessment of treatment efficacy, using the post-treatment biopsy as a reference. Briefly, histopathology proved 29% responders (decreasing stage), 24% non-responders (increasing stage), but was not conclusive in 47% of cases (no change in stage). In contrast, AI identified 53% of cases as responders (congruous decrease of both EC and EnC), 23% non-responders (congruous increase of both EC and EnC) and 24% as not conclusive (discordant ECA and EnC). Figure 6 illustrates the impact of AI on the evaluation of fibrosis modification due to treatment.

## 4 Discussion

Histopathological characterization of fibrosis is the only parameter that correlates with the evolution of steatohepatitis (5–7). A reduction in fibrosis is one of the goals of the treatments for steatohepatitis, as well as one of the parameters used by the FDA for drug approval (25). Nonetheless, the evaluation of fibrosis modification under the microscope has some limitations. First, the assessment is a highly subjective process: numerous studies have demonstrated good but not exceptional agreement even among expert hepato-pathologists (26). Then, the system in use classifies into defined categories, similar to impermeable compartments, a process, the appearance, and modulation of fibrosis, which in nature appears as continuous (27). Finally, liver fibrosis remodeling is a slow event that might not be depicted properly by the few categories of current staging systems. To overcome these limitations, some studies started using AI to quantify fibrosis in a more objective way and as a continuous parameter, or to identify specific parameters that might not be defined by histopathology, for example the entropy of collagen fibers (13, 16, 28, 29).

The results of the present study, conducted on MASH biopsy confirm that AI-based evaluation characterizes at the deepest level, compared to classic histopathological evaluation, the fibrotic process occurring in patients with MASH. In particular, we showed that cases consistently labeled as belonging to the same histopathological stage might show significant differences in collagen amount. Thus, cases classified as F3 might present values identical to a F2- or to a F4- cases. This infinitesimal evaluation of the collagen amount likely represents the result of the multi-resolution approach we adopted in the study. Indeed, the AI we designed combined features related to texture and intensity obtained at two clearly separated spatial scales: single pixel and ROI (32x32pixel). Interestingly, Taylor-Weiner et al. (16), using a similar approach, a deep-learning method able to measure the amount and distribution of fibrosis across the whole biopsy at different resolutions, proved a significant heterogeneity across patients with the same stage. A significant heterogeneity across patients with the same stage was also proved by Wang et al. (29) and Forlano et al. (13) using Collagen Proportionate Area (CPA). Interestingly enough the values of CPA reported by these two studies for specific MASH stages (F2: 3.3 and 2.1%; F3: 6.8 and 5.5%; F4: 12.4 and 11.1%) overlap those of ECA in our study (F2: 2.6%; F3: 5.7% and F4: 10.9%).

The present study also confirmed that AI allows a more accurate identification of minimal changes conditioned by the therapy in MASH patients. In fact, while histopathology identified stage changes in 53% of cases, the congruous combination of two AI features representing the amount and entropy of collagen highlighted the presence of changes in 76% of the cases. These findings are strictly in keeping with the few similar data available in the literature. Naoumov et al. (15) proved a change in stage in 97% of cases using digital quantification on SHG as compared to 40% detected by histopathology. In specific, stage regression was observed in 69% of F2 and F3 cases treated with the optimal dose of drug. Taylor-Wayner et al. (16) calculated 27 and 40% of responders using, respectively, a rigorous or less-stringent thresholds as compared to none detected by histopathology. In our series, AI proved a stage regression in 53% of cases; histopatology in 29%; weight loss greater than 10%, a clinical feature that has been associated with improvements in liver fibrosis in MASH (24), in 12%. We also showed that the reduction in absolute terms of collagen was frequently accompanied by a reduction of entropy of collagen (EnC). This latter is a textural parameter that translates the randomness of Sirius Red optical density values with respect to its neighborhood in terms of the intensity distribution. Low entropy values correspond to more uniform and homogeneous image thus to more grouped collagen fibers. Interestingly enough, our findings overlap the description of regressing fibrosis reported by Sanyal et al. (7) characterized by the narrowing or even disappearance of the fibrotic bridge; as well as that described by Naoumov et al. (15) showing that after treatment septa became thin and compact, with sharp borders. Once inserted into this framework, our results, although limited to a small series of post treatment biopsy, do not appear random but rather representative of the important role played by AI in highlighting changes caused by medical therapy.

Finally, we showed that some post treatment liver biopsy totally unchanged under the microscope, have only minimal changes also in the parameters extracted by AI. This result mirrors those of a SHG study showing that fibrosis regression starts in a piecemeal manner (15). Interestingly enough, a recent paper, based on the spatial information returned by SHG proved, in a murine model of steatohepatitis, that this minute fibrosis regression is induced by a reduction in food amount (30).

In conclusion, our data confirm that AI allows a better characterization of the fibrosis process by its true, continuous, and non-categorical nature. Of clinical importance is also the possibility, guaranteed by AI algorithms, of identifying among the cases that the pathologist would have judged as unchanged, those in which there was actually a response to treatment. Finally, it does not appear irrelevant to observe that there is a certain percentage of cases where the objective parameters generated by AI highlight an initial process of modification of the fibrosis. Taken together, the intrinsic possibilities of AI, pave the way for renewed staging system, where the information generated by pathological description (site of fibrosis; formation of septa) would benefit of an integration with data generated by AI.

## Data Availability

The original contributions presented in the study are included in the article/Supplementary material, further inquiries can be directed to the corresponding authors.
