# Supplementary material for: Role of artificial intelligence in staging and assessing of treatment response in MASH patients
Source: Front Med (Lausanne). 2024 Oct 21;11:1480866. doi: 10.3389/fmed.2024.1480866 (PMC11532183; doi:10.3389/fmed.2024.1480866)
Supplement: Supplementary file 1 [file Data_Sheet_1.pdf]

Supplementary Material

1 Supplementary Methods

1.1 WSI Acquisition and (Digital) Handling / WSI Imaging and Conversion

Whole-Slide Images (WSIs) are acquired through the Ultra Fast Scanner (Philips, Netherlands) and stored in the proprietary iSyntax format. Imaging is performed with an Olympus 40x air Objective (NA = 0.75, Plan Apo) with a pixel-size of 0.25 μm. The libraries and scripts in the Philips Pathology SDK 2.0 are exploited to convert the images from the iSyntax to the Tagged Image File Format (TIFF) while simultaneously anonymizing the slides. The resulting TIFF files are compressed with the JPEG image scheme with at least four different pyramidal layers. Each layer in the pyramidal image is composed of identically sized square image tiles (e.g., 1024x1024 for the finest/first layer). WSI are managed through libraries within the Blocked Processing Toolbox, a component of the MATLAB Suite's Image Processing Library, designed to optimize tasks on individual tiles (The MathWorks Inc. MATLAB version: R2023b, Natick, Massachusetts, 2023).

1.2 Preprocessing

In our pipeline, each pixel of the image is converted from the RGB colorspace into an optical density (OD) triplet. The optical density (OD_i) for each color channel (i.e., R, G, B) is determined by the following relation:

$$OD_i = -log\left(\frac{I_i}{I_0}\right) \qquad (i = R, G, B)$$

where I_0 represents the background RGB contribution and its estimate is performed either automatically, by taking the modal RGB value of the fourth pyramid level, or by manually selecting a TIFF tile without tissue.

The tissue was separated from the background through a semi-automatic thresholding procedure, achieved by applying Otsu's Thresholding (Otsu, 1979)to the sum of the OD triplets within a selected region as shown in Supplementary Figure 1. Pixels below this threshold are disregarded in further analyses. Moreover, tiles covered by less than 1% of tissue area were not considered in the image analysis.

1.3 SR Segmentation

Stain vector deconvolution was performed to extract the Sirius Red contribution. To derive the stain vectors and stain matrix, we employed a custom script, implementing Macenk's algorithm (Macenko et al., 2009). The stain vector computation was performed on at least 20 representative random selected tiles. After stain matrix estimate, stain deconvolution has been performed on the whole image. In this way, we obtain the Sirius Red OD contribution. In order to segment the collagen deposition, we employed Otsu thresholding on the OD values obtained from the deconvolution process.

1.4 Quantification

After segmentation, we performed quantification of the collagen structure by employing a multi-resolution approach. This involves the extraction of both intensity and textural features at two different spatial scales: at the pixel level and within re-binned Regions of Interest (ROIs).

Pixel-wise quantification was obtained from the 2nd level of the image pyramid (1 pixel = 0.5 μm) to minimize any potential artifacts arising from the JPEG lossy compression scheme. We further divided this pyramid level into discrete 32x32 pixels regions (16x16 μm^2) to estimate the local collagen properties. A comprehensive list of all the features is provided in Supplementary Table 1.

Estimated Collagen Area (ECA) is computed as a fraction of collagen pixels (Sirius Red positive) over the total number of pixels representing the tissue section. In the main text, ECA is reported as a percentage (0-100%) referring to the entire tissue section.

Entropy of Collagen (EnC) is a textural parameter which encodes randomness of the values in the selected 32x32 pixels ROI. It is defined as:

$$EnC = -\sum_{k} p_k log(p_k)$$

where p_k is is the probability associated with a specific gray level k. Entropy ranges between 0 and log N where N is the number of gray-levels in the input image (Shannon, 1948).

Supplementary Table 2 shows, for each patient, the results of those parameters which, according to the statistical analysis, better illustrates the difference of fibrosis.

1.5 Statistical Analysis

Statistical analyses were performed using Prism GraphPad software v. 8.0.2 (GraphPad Software, San Diego, CA, USA). Data were analyzed using multiple comparisons of one-way ANOVA corrected by a Tukey post hoc test. A p-value less than 0.05 was considered significant.

2 Supplementary Figures and Tables

2.1 Supplementary Figures

Supplementary Figure 1. Otsu thresholding procedure to separate tissue from the background.

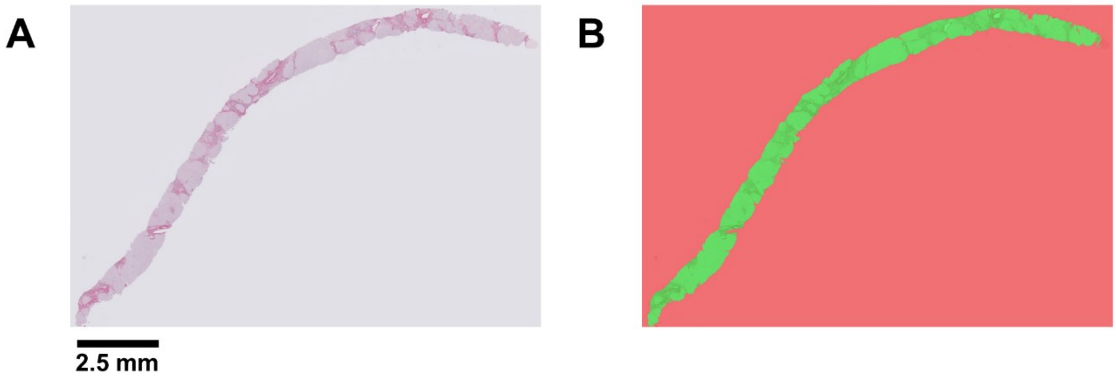

Caption. A semi-automatic thresholding procedure to select liver tissue from the slide background. A) The acquired original RGB WSI. B) Pre-processing of the same image in panel A. Otsu's Thresholding was employed to separate the liver tissue (green, tiles processed by the pipeline) from background (red, tiles disregarded during the analysis).

Supplementary Figure 2. EnC heterogeneity in F2- and F3- stage cases.

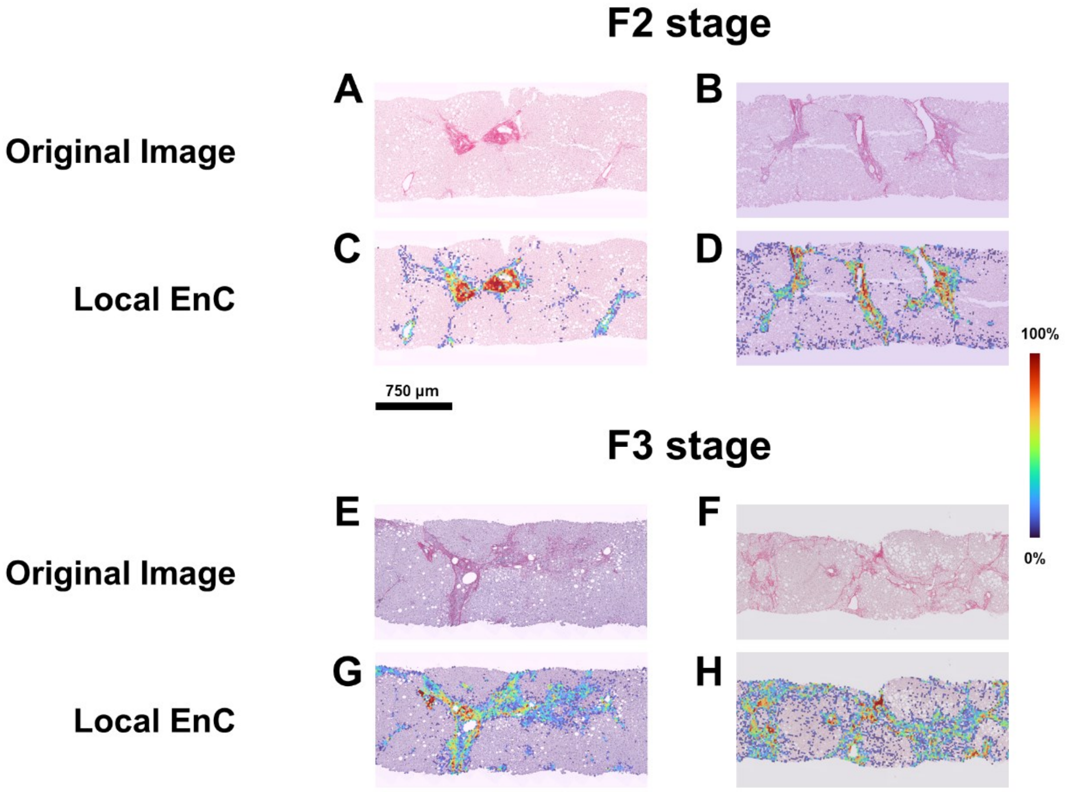

Caption. This figure shows the results of collagen entropy evaluation generated by the AI-algorithm in the same cases of Figure 4. Also for this parameter, the AI reveals differences among cases labeled as homogenous at histopathological level. Cases diagnosed as F2 show respectively EnC=0.84±0.01 (cases A, heatmap C) and EnC=0.99±0.01, (cases B, heatmap D) while cases diagnosed as F3 show EnC=1.21±0.01 (case E, heatmap G) and EnC=1.59±0.01 (case F, heatmap H). The reported EnC values refer to the entire tissue section.

2.2 Supplementary Tables

Supplementary Table 1. List and description of all the extracted features from the WSI.

Number	Feature	Description
1	Positive Pixel Count	Number of pixels which are considered belonging to Sirius Red positive areas
2	Total Pixel Count	Number of pixels which are considered belonging to the tissue
3	**Positive Fraction (PW) = ECA**	Ration between "Positive Pixel Count" and "Total Pixel Count". Reported into a percentage it represents the Estimated Collagen Area (ECA).
4	OD Sum (PW)	Sum over all the positive pixels of the Sirius Red OD contributions.
5	AVG OD of the Positive Pixels (PW)	Average value, over all the positive pixels, of the Sirius Red OD contributions. Also "OD Sum (PW) / Positive Pixel Count
6	AVG OD of the Tissue Pixels (PW)	Average value, over all the tissue pixels, of the Sirius Red OD contributions. Also "OD Sum (PW) / Total Pixel Count
7	OD Sum (PW) * ECA (PW)	Sum over all the positive pixels of the Sirius Red OD contributions multiplied by the Positive Fraction (ECA). Also "OD Sum * Positive Fraction"
8	OD Sum (PW) / ECA (PW)	Sum over all the positive pixels of the Sirius Red OD contributions divided by the Positive Fraction (ECA). Also "OD Sum / Positive Fraction"
9	OD Sum (AVG)	These features are the same as the ones already listed before, but are calculated on 32x32 pixels ROIs. For every feature we computed the average value and its standard deviation over the whole tissue section.
10	OD Sum (SE)	
11	Estimated Collagen Area (AVG)	
12	Estimated Collagen Area (SE)	
13	AVG OD of the Positive Pixels (AVG)	
14	AVG OD of the Positive Pixels (SE)	
15	AVG OD of the Tissue Pixels (AVG)	
16	AVG OD of the Tissue Pixels (SE)	
17	AVG OD * ECA (AVG)	
18	AVG OD * ECA (SE)	
19	AVG OD / ECA (AVG)	
20	AVG OD / ECA (SE)	
21	Contrast (AVG)	These features are estimated on 32x32 pixels ROIs using the Grey Level Co-Occurrence Matrix (GLCM). For every feature we computed the average value and its standard deviation over the whole tissue section. They describe the local textural properties of the collagen deposition (Haralick, Shanmugam and Dinstein, 1973).
22	Contrast (SE)	
23	Correlation (AVG)	
24	Correlation (SE)	
25	Energy (AVG)	
26	Energy (SE)	
27	Homogeneity (AVG)	
28	Homogeneity (SE)	
29	**Entropy (AVG) = EnC**	Encodes randomness of the Sirius Red OD contributions of a 32x32 pixels ROI. We computed the average value and its standard deviation over the whole tissue section.
30	Entropy (SE)	
31	ECA * EnC (PW)	Product between Estimated Collagen Area and Entropy of Collagen.
32	EnC / ECA (PW)	Ratio between Entropy of Collagen and Estimated Collagen Area.
33	ECA / EnC (PW)	Ratio between Estimated Collagen Area and Entropy of Collagen.

Supplementary Table 2. Results of the most significant AI-based features for each case of the series.

Fibrosis Stage	ECA*EnC	%ECA	Local_ECA	ECA/EnC	EnC	Energy	Homogeneity	EnC/ECA	OD Sum * ECA	Contrast	Correlation	Mean OD of the Tissue Pixels	OD Sum / ECA	Mean OD of the Positive Pixels
3	0.152	9.0%	24.8%	0.053	1.70	0.61	0.82	18.9	26.6	9.1	0.27	0.056	2.22	0.21
4	0.135	8.6%	25.1%	0.055	1.56	0.65	0.83	18.2	21.0	14.6	0.30	0.051	1.79	0.17
3	0.087	6.3%	22.6%	0.046	1.38	0.70	0.85	21.9	17.5	11.3	0.28	0.043	1.96	0.16
3	0.039	3.4%	17.1%	0.030	1.14	0.73	0.88	33.3	9.2	5.8	0.27	0.025	1.81	0.13
3	0.123	7.1%	24.8%	0.041	1.73	0.61	0.81	24.3	22.0	9.7	0.31	0.045	1.66	0.16
3	0.041	4.6%	14.0%	0.053	0.88	0.79	0.91	19.0	4.7	3.4	0.24	0.018	1.77	0.11
1a	0.007	0.7%	16.6%	0.006	1.12	0.75	0.88	170.1	15.9	11.7	0.27	0.041	3.27	0.21
3	0.043	4.2%	16.1%	0.040	1.04	0.76	0.89	24.7	8.8	4.9	0.25	0.022	1.74	0.11
3	0.051	5.8%	14.0%	0.066	0.87	0.80	0.91	15.1	7.8	5.1	0.22	0.022	2.10	0.13
2	0.032	3.9%	13.7%	0.046	0.84	0.80	0.91	21.8	7.5	5.2	0.22	0.022	2.24	0.13
4	0.187	10.2%	28.0%	0.056	1.84	0.59	0.80	18.0	23.8	12.8	0.32	0.049	1.34	0.15
1a	0.010	1.1%	13.0%	0.013	0.86	0.80	0.92	75.8	5.5	2.7	0.24	0.017	2.01	0.11
3	0.046	5.3%	12.6%	0.062	0.86	0.79	0.92	16.1	4.7	3.9	0.24	0.019	2.10	0.14
2	0.013	1.2%	15.6%	0.011	1.10	0.75	0.88	91.9	14.4	9.8	0.27	0.039	3.55	0.21
2	0.012	1.7%	11.2%	0.023	0.73	0.83	0.93	43.0	4.6	2.6	0.22	0.016	2.28	0.12
1c	0.019	1.4%	19.9%	0.011	1.32	0.70	0.86	93.1	13.5	9.3	0.29	0.037	2.02	0.16
2	0.014	2.6%	8.6%	0.046	0.56	0.87	0.95	21.8	2.5	1.6	0.18	0.011	2.56	0.12
3	0.063	4.8%	18.7%	0.036	1.32	0.68	0.86	27.5	8.8	6.1	0.29	0.027	1.47	0.13
4	0.218	10.3%	31.7%	0.048	2.12	0.52	0.76	20.7	23.9	15.3	0.31	0.053	1.05	0.15
3	0.073	6.5%	16.0%	0.058	1.12	0.73	0.88	17.4	9.8	5.9	0.26	0.027	2.31	0.15
3	0.040	3.9%	15.5%	0.038	1.02	0.75	0.89	26.2	8.3	6.1	0.25	0.025	2.09	0.13
3	0.075	6.5%	17.3%	0.056	1.16	0.74	0.87	17.8	12.6	10.2	0.25	0.031	2.16	0.15
2	0.088	6.6%	19.7%	0.050	1.33	0.70	0.85	20.1	16.5	12.3	0.27	0.037	1.99	0.15
2	0.017	2.0%	15.3%	0.022	0.89	0.79	0.91	45.4	6.7	5.6	0.24	0.024	2.04	0.14
1c	0.012	1.0%	16.6%	0.008	1.21	0.70	0.87	125.8	9.4	8.4	0.28	0.028	1.99	0.15
2	0.013	1.5%	12.6%	0.016	0.89	0.79	0.91	61.4	6.7	5.9	0.24	0.021	2.58	0.14
3	0.142	9.6%	24.8%	0.065	1.48	0.65	0.83	15.5	15.2	11.7	0.28	0.041	1.37	0.15
3	0.113	8.0%	23.9%	0.056	1.42	0.68	0.85	17.8	17.8	12.7	0.27	0.041	1.52	0.14
2	0.018	2.3%	11.1%	0.031	0.75	0.81	0.92	32.1	4.1	4.0	0.23	0.017	2.35	0.13
1a	0.017	2.0%	13.2%	0.023	0.86	0.79	0.91	43.4	9.2	8.1	0.23	0.029	3.30	0.19
2	0.025	2.7%	13.0%	0.028	0.95	0.78	0.89	35.2	12.3	10.8	0.22	0.029	3.52	0.18
2	0.017	1.3%	19.5%	0.011	1.27	0.71	0.86	94.5	17.5	11.3	0.31	0.045	2.52	0.20
3	0.061	3.8%	21.0%	0.024	1.59	0.64	0.82	41.5	26.6	22.8	0.29	0.062	3.22	0.26
4	0.191	12.2%	21.0%	0.078	1.56	0.63	0.81	12.8	20.6	25.4	0.23	0.055	2.61	0.24
2	0.023	3.1%	12.4%	0.042	0.75	0.82	0.92	23.7	5.7	4.3	0.22	0.023	2.68	0.16
2	0.009	0.9%	13.8%	0.009	0.99	0.77	0.89	111.5	9.6	6.9	0.26	0.031	3.37	0.20
3	0.200	9.8%	28.4%	0.048	2.04	0.56	0.77	20.7	34.3	20.9	0.33	0.064	1.82	0.19
3	0.068	4.8%	20.8%	0.034	1.42	0.68	0.84	29.6	19.0	13.5	0.28	0.044	2.28	0.17
2	0.025	1.6%	22.7%	0.011	1.50	0.65	0.84	91.7	16.0	9.2	0.33	0.038	1.51	0.14
3	0.063	5.8%	16.5%	0.054	1.08	0.77	0.89	18.6	12.9	8.8	0.24	0.031	2.28	0.15
3	0.075	5.9%	19.2%	0.045	1.29	0.71	0.86	22.0	14.2	10.4	0.26	0.036	2.06	0.16
3	0.038	3.8%	16.9%	0.038	1.00	0.78	0.90	26.3	10.9	7.2	0.25	0.028	1.92	0.13
2	0.041	5.5%	11.4%	0.075	0.74	0.82	0.92	13.4	6.8	6.0	0.21	0.023	3.22	0.18
3	0.053	4.2%	18.5%	0.033	1.26	0.71	0.86	30.0	14.6	8.2	0.30	0.035	2.29	0.16
2	0.017	1.7%	14.9%	0.017	0.99	0.77	0.90	59.3	7.5	3.5	0.25	0.021	2.00	0.12
3	0.061	6.3%	13.6%	0.066	0.96	0.76	0.90	15.1	6.6	6.6	0.25	0.027	2.64	0.18
3	0.040	3.2%	18.5%	0.026	1.25	0.72	0.87	38.9	14.8	6.0	0.28	0.032	2.04	0.14
4	0.257	13.4%	28.6%	0.069	1.92	0.58	0.78	14.4	26.5	18.4	0.29	0.053	1.53	0.16
2	0.037	3.8%	15.5%	0.039	0.99	0.78	0.90	25.9	9.1	6.2	0.24	0.026	2.10	0.14
1b	0.023	3.0%	10.8%	0.040	0.76	0.81	0.92	25.0	5.7	7.2	0.22	0.024	3.50	0.19
3	0.027	2.8%	15.0%	0.029	0.97	0.77	0.90	34.7	8.5	5.5	0.26	0.025	2.19	0.14
2	0.037	3.7%	14.8%	0.036	1.01	0.76	0.89	27.5	10.0	6.7	0.26	0.026	2.34	0.15
1a	0.006	0.9%	12.1%	0.015	0.63	0.85	0.95	68.4	2.5	1.0	0.25	0.012	1.66	0.09
2	0.014	1.5%	12.1%	0.017	0.89	0.77	0.89	57.4	14.2	20.1	0.23	0.065	8.98	0.50
3	0.121	9.9%	19.2%	0.082	1.21	0.72	0.87	12.2	12.8	9.3	0.29	0.035	1.90	0.16
1c	0.025	2.3%	14.5%	0.021	1.10	0.74	0.86	48.3	26.0	30.8	0.23	0.085	8.85	0.54
2	0.016	1.4%	16.5%	0.012	1.12	0.74	0.88	80.5	11.8	7.6	0.27	0.031	2.43	0.16
1c	0.010	1.1%	13.0%	0.013	0.85	0.80	0.91	75.3	9.7	7.7	0.24	0.029	3.57	0.19
3	0.075	5.1%	22.0%	0.035	1.46	0.68	0.84	28.4	20.7	12.9	0.30	0.043	1.90	0.16
3	0.054	5.2%	19.1%	0.050	1.04	0.75	0.89	20.0	9.7	7.8	0.26	0.033	1.75	0.15
3	0.049	5.0%	16.0%	0.051	0.98	0.78	0.90	19.5	9.7	8.5	0.24	0.028	2.04	0.14

Supplementary Table 3. Estimated Collagen Area (mean; Inter Quartile Range – IQR; standard deviation of the mean) for each pathological stage.

**ECA**	**1a**	**1b***	**1c**	**2**	**3**	**4**
**ECA Mean**	1.2	3.05	1.4	2.6	5.7	10.9
**IQR**	1.1	-	1.1	2.2	2.3	3.4
**Std. deviation of Mean**	0.3	-	0.3	0.4	0.4	0.8

(*there was only one case 1b)

Supplementary Table 4. Entropy of Collagen (mean; Inter Quartile Range – IQR; standard deviation of the mean) for each pathological stage of NASH in the present series.

**EnC**	**1a**	**1b***	**1c**	**2**	**3**	**4**
**Mean**	0.9	0.76	1.1	0.96	1.24	1.8
**IQR**	0.4	-	0.4	0.4	0.4	0.5
**Std. deviation of Mean**	0.1	-	0.1	0.05	0.06	0.1

(*there was only one case 1b)

Supplementary Table 5 a and b: Clinical features of the series with paired biopsies.

Patient	Age	Sex	Weight (Kg)	Height (cm)	BMI	Hypertension	Diabetes	Dyslipidemia	AST levels (U/L)	ALT levels (U/L)	Platelets (10^9/mm^3)
N1	52	M	95	165	34.9	Yes	No	Yes	63	40	186
N2	46	M	97	172	32.8	No	No	Yes	31	25	287
N3	47	F	92	156	37.8	Yes	No	No	29	28	298
N4	68	M	87	165	32	No	No	Yes	30	34	234
N5	50	M	102	191	28	No	No	Yes	83	45	257
N6	57	M	90	172	30.4	No	Yes	Yes	64	39	254
N7	66	M	95	183	28.4	Yes	No	No	93	67	202
N8	62	F	75	160	29.3	Yes	Yes	No	79	43	119
N9	71	F	67	155	27.9	No	No	Yes	46	39	253

Supplementary Table 5a: Demographic and clinical characteristics at baseline of 9 patients undergoing paired liver biopsies before and after treatment in a clinical trial for MASH (study cohort)

Patient	Age	Sex	Weight (Kg)	Height (cm)	BMI	Hypertension	Diabetes	Dyslipidemia	AST levels (U/L)	ALT levels (U/L)	Platelets (10^9/mm^3)
N1	56	M	103	175	33.6	Yes	Yes	Yes	66	117	336
N2	60	M	111	175	36.2	Yes	Yes	No	32	40	300
N3	46	M	74	178	23.3	Yes	No	Yes	27	46	320
N4	66	M	85	163	32	Yes	No	Yes	47	87	286
N5	64	F	70	162	26.5	No	No	Yes	42	75	310
N6	24	M	109	192	29.5	No	No	Yes	70	278	157
N7	51	M	83	168	29.4	Yes	Yes	No	71	142	149
N8	54	M	86	168	30.5	Yes	Yes	No	71	93	199

Supplementary Table 5b: Demographic and clinical characteristics at baseline of 8 patients undergoing paired liver biopsies before and after treatment in a clinical trial for MASH (validation cohort)

3 SUPPLEMENTARY REFERENCES

Haralick, R.M., Shanmugam, K. and Dinstein, I. (1973) ‘Textural Features for Image Classification’, IEEE Transactions on Systems, Man, and Cybernetics, SMC-3(6), pp. 610–621. Available at: https://doi.org/10.1109/TSMC.1973.4309314.

Macenko, M. et al. (2009) ‘A method for normalizing histology slides for quantitative analysis’, in 2009 IEEE International Symposium on Biomedical Imaging: From Nano to Macro. IEEE, pp. 1107–1110. Available at: https://doi.org/10.1109/ISBI.2009.5193250.

Otsu, N. (1979) ‘A Threshold Selection Method from Gray-Level Histograms’, IEEE Transactions on Systems, Man, and Cybernetics, 9(1), pp. 62–66. Available at: https://doi.org/10.1109/TSMC.1979.4310076.

Shannon, C.E. (1948) ‘A Mathematical Theory of Communication’, Bell System Technical Journal, 27(3), pp. 379–423. Available at: https://doi.org/10.1002/j.1538-7305.1948.tb01338.x.

The MathWorks Inc. MATLAB version: R2023b, Natick, Massachusetts (2023). Available at: https://www.mathworks.com.
